# Neonatal Symptoms in Pediatric Idiopathic Growth Hormone Deficiency: Prevalences and Insights

**DOI:** 10.3390/children12040430

**Published:** 2025-03-28

**Authors:** Giorgio Sodero, Donato Rigante, Clelia Cipolla

**Affiliations:** 1 Department of Life Sciences and Public Health, Fondazione Policlinico Universitario A. Gemelli, IRCCS, 00168 Rome, Italy; 2 Pediatric Department, Perrino Hospital, 72100 Brindisi, Italy; 3 Pediatric Endocrinology Unit, Perrino Hospital, 72100 Brindisi, Italy; 4 Periodic Fever and Rare Diseases Research Centre, Università Cattolica del Sacro Cuore, 00168 Rome, Italy

**Keywords:** growth hormone, growth hormone deficiency, pediatric endocrinology, neonatal hypoglycemia

## Abstract

Background: Growth hormone deficiency (GHD) is one of the primary endocrine-related causes of short stature in pediatric patients; while neonatal GHD symptoms are well-documented in populations with known genetic and/or organic causes, their exact prevalences in pediatric patients categorized as having idiopathic GHD remains unclear. Materials and Methods: We retrospectively analyzed the medical records of patients with idiopathic GHD followed at the Pediatric Endocrinology Unit of the Fondazione Policlinico Universitario A. Gemelli IRCCS starting from January 2010. We analyzed information from 190 patients with idiopathic GHD and examined the prevalences of the most common neonatal signs and symptoms of neonatal GHD. We also included an age- and sex-matched control group that consisted of patients without a confirmed diagnosis of GH deficiency to assess significant differences in the frequencies of neonatal symptoms between the two cohorts. Results: Regarding neonatal GHD symptoms, the prevalence was the highest for hypoglycemia (n = 53, 27.9%), which was managed through the intravenous administration of glucose in 21 out of 53 cases. Prolonged jaundice that lasted more than 5 days was observed in 37 patients (19.5%) and required phototherapy in 20 out of 37 patients, while exchange transfusion was not performed in any patient. Hyperglycemia and feeding difficulties (n = 17, 8.9%) were less frequent, while the other symptoms were relatively rare. Compared with the control group, the prevalence of hypoglycemia was significantly higher in the GHD patient group (*p*-value = 0.000016). Conclusions: In our cohort of pediatric patients with idiopathic GHD, the prevalences of neonatal signs and symptoms of GHD was low, except for neonatal hypoglycemia observed in 27.9% of the analyzed patients. Although these are not specific signs of idiopathic GHD, it is beneficial to investigate this information in the medical history during the clinical assessment of the child.

## 1. Introduction

Growth hormone deficiency (GHD) is one of the primary endocrine-related causes of short stature in pediatric patients [1]. Growth hormone (GH) replacement therapy represents the main therapeutic approach for this disorder and is also utilized in other clinical conditions with organic hormone deficiency (caused by the destruction or compression of the hypothalamic–pituitary region due to space-occupying processes, inflammation, or trauma) [2] or in patients with genetic syndromes, even in the absence of a confirmed hormonal deficiency [3].

Certain forms of GHD can manifest as early as the neonatal period [1]; these severe cases may be associated with underlying genetic defects or midline abnormalities and often present with nonspecific symptoms, such as persistent hypoglycemia or prolonged jaundice [4]. In these cases, the diagnosis of GHD is challenging and often missed in the early stages, except in cases associated with multiple pituitary hormone deficiencies [1,4].

Idiopathic GHD is a condition characterized by the insufficient secretion or production of growth hormone by the pituitary gland, leading to impaired growth and development in both children and adults [1]. In cases of children with idiopathic GHD, the cause is unknown, meaning no specific underlying pathology, such as genetic defects or tumors, can be identified [2]. Diagnosis typically involves stimulation tests to assess GH secretion. A result of less than 8 ng/mL during these stimulation tests is generally considered indicative of GHD, as normal GH levels typically exceed this threshold in healthy individuals [1]. In Italy, the diagnosis of GHD requires at least two negative stimulation tests, meaning that GH levels below 8 ng/mL must be observed in two separate tests to confirm the deficiency. Blood samples were collected at baseline (time 0) and at 15, 30, 60, 90, and 120 min post-stimulation, which resulted in a total of six samples for GH measurement. GHD was confirmed if the GH levels were below 8 ng/mL in both tests. In cases where one test was positive and the other negative, patients were monitored over time. If a further deceleration in growth rate and/or a worsening of height percentile was observed, a third stimulation test could be performed to confirm or rule out the diagnosis of idiopathic GHD.

While neonatal GHD symptoms are well-documented in populations with known genetic and/or organic causes [1,2], their exact prevalences in pediatric patients categorized as having idiopathic GHD remains unclear. This uncertainty is often due to the incomplete or retrospective nature of neonatal information, typically analyzed years after birth.

In this short study, we examined the prevalences of the most common neonatal signs and symptoms of GHD in a cohort of children with a confirmed diagnosis of idiopathic GHD.

## 2. Materials and Methods

We retrospectively analyzed the medical records of patients with idiopathic GHD followed at the Pediatric Endocrinology Unit of the Fondazione Policlinico Universitario A. Gemelli IRCCS starting from January 2010. Ethics committee approval was not obtained because the General Authorization to Process Personal Data for Scientific Research Purposes (Authorization No. 9/2014) states that retrospective archival studies using ID codes that prevent the direct tracing of data to the patient do not require formal ethics approval. Additionally, the information extracted from the medical records pertains to the routine therapeutic management of patients with GHD. All patients were re-contacted to authorize access to their clinical records by completing a written informed consent form; for adult subjects, consent was provided by the patients themselves.

We also included an age- and sex-matched control group that consisted of patients followed up at the Pediatric Endocrinology Unit of the Fondazione Policlinico Universitario A. Gemelli IRCCS without a confirmed diagnosis of GH deficiency. The control group was carefully selected to be age- and sex-matched to the study cohort to ensure comparable demographic characteristics; all control subjects were born in the same period to minimize potential confounding factors related to age and birth cohort effects. We compared the frequencies of neonatal signs associated with GHD between the two groups to assess their prevalences and potential diagnostic relevance.

The extracted data were collected in an Excel database and included information on the birth weight, sex, age, and auxological parameters at the time of GHD diagnosis; baseline IGF-1 levels; and information on the most common neonatal signs and symptoms of GHD [4]: hypo- and hyperglycemia, cryptorchidism, micropenis (<2 standard deviations for age), prolonged jaundice (lasting more than 5 days), cholestasis signs, neonatal hypotonia, and feeding difficulties in the first days of life.

According to our center protocol, neonatal hypoglycemia refers to abnormally low blood glucose levels in a newborn, typically defined as a plasma glucose concentration below 40 mg/dL (2.2 mmol/L) in the first 24 h of life, or below 45 mg/dL (2.5 mmol/L) thereafter. Hypoglycemia in neonates can lead to a range of symptoms, including poor feeding; lethargy; seizures; and in severe cases, long-term neurological damage if not promptly treated. Neonatal hyperglycemia refers to abnormally high blood glucose levels in a newborn, typically defined as a plasma glucose concentration greater than 150 mg/dL (8.3 mmol/L). Hyperglycemia in neonates can occur due to factors such as stress, infection, or underlying metabolic disorders, and may require management to prevent complications such as dehydration, electrolyte imbalances, and potential long-term health issues.

Categorical variables are expressed as numbers, and continuous variables are expressed as means and standard deviations. For continuous variables, the Shapiro–Wilk test was used to determine whether the distribution was normal. Groups of continuous variables were compared with Student’s *t*-test (normally distributed data).

## 3. Results

Overall, we analyzed information from 190 patients with idiopathic GHD; the study sample had a mean age of 10.9 years (±3.1). Males comprised the majority of the cohort, with 140 individuals, which accounted for 73.7% of the total population. The average birth weight was 3.08 kg (0.49 standard deviation). All patients included in our study group were born at term (>37 weeks of gestational age) and had an appropriate birth weight for gestational age (AGA). All patients diagnosed with GHD underwent daily growth hormone therapy, with a dosage that ranged from 0.025 to 0.035 mg/kg/day, as recommended by our national guidelines. The bone age was delayed by more than 2 years in 120 out of 190 patients (63.2%).

In all the patients in our study group, the diagnosis of GHD was confirmed through the execution of two growth hormone stimulation tests, both of which yielded pathological results (GH peak < 8 ng/mL). Given the heterogeneity of the tests performed and the requirement, according to our national guidelines, to conduct at least two tests for diagnosis, we did not perform a comparative analysis between the GH peak observed during the various tests and the frequencies of neonatal signs and symptoms.

Regarding neonatal GHD symptoms, the prevalence was the highest for hypoglycemia (n = 53, 27.9%), which was managed through the intravenous administration of a glucose solution in 21 out of 53 cases. Prolonged jaundice that lasted more than 5 days was observed in 37 patients (19.5%) and required phototherapy in 20 out of 37 patients, while an exchange transfusion was not performed in any patient. Hyperglycemia and feeding difficulties (n = 17, 8.9%) were less frequent, while the other symptoms were relatively rare. More information is provided in Table 1.

We subsequently compared the frequencies of various neonatal symptoms reported in our cohort of patients with GHD with the prevalences of the same signs in our control group, which consisted of healthy pediatric patients. No significant differences were observed for most signs; however, the prevalence of hypoglycemia was found to be significantly higher in the GHD patient group (*p*-value = 0.000016). More information is provided in Table 1.

## 4. Discussion

In our short communication, we analyzed the prevalences of neonatal signs of GHD in a large retrospective cohort of children. The descriptive analysis of the signs and symptoms of GHD revealed that the only frequent neonatal sign of growth hormone deficiency in our cohort of children with idiopathic GHD was hypoglycemia. The comparative analysis with a group of pediatric patients without a GHD diagnosis highlighted that the prevalence of hypoglycemia is a clinically relevant indicator of neonatal GHD. Therefore, hypoglycemia should be considered in the potential endocrine follow-up of these patients.

Neonatal hypoglycemia may be closely related to the presence of neonatal GH deficiency due to the strong connection between growth hormones and glucose metabolism [1], which begins in the early stages of pregnancy and continues throughout life [5]. Typically, patients undergoing GH therapy, in addition to the regular monitoring of IGF-1, should periodically have their blood glucose levels monitored to assess any metabolic imbalances that result from the treatment [1]. The causal association between GHD and neonatal hypoglycemia can be easily demonstrated by measuring the GH levels during a hypoglycemic episode [6,7,8]; however, in clinical practice, this is rarely performed in the absence of a strong clinical suspicion of hypopituitarism, as the other causes of neonatal hypoglycemia are more common.

Hypoglycemia associated with GHD is often an exacerbation of complex genetic defects or anatomical alterations of the hypothalamic–pituitary axis [9,10,11]; in a cohort of patients with hypoglycemia treated with growth hormone due to the suspicion of hypopituitarism, it was found that structural lesions of the hypothalamic–pituitary area or midline anatomical facial defects were present in approximately one-third of the neonates [6]. However, a history of variable and nonspecific hypoglycemia is sometimes reported by patients diagnosed with confirmed or suspected GHD [1].

Regarding other neonatal signs and symptoms of GHD, although they are reported in the literature as potential early indicators of GHD, they were not found to be associated with the diagnosis of idiopathic GH deficiency in our study cohort. However, it is possible that modifying our inclusion criteria—such as incorporating patients with genetically determined GH deficiency with early onset—could yield different statistical results.

Our short study had some limitations, as it analyzed data retrospectively from a single center. Many of the patients classified as having idiopathic GHD may have had undiagnosed mild genetic forms that could explain the paucisymptomatic neonatal onset, as genetic analysis is not part of the first-line investigations for patients with a growth hormone deficiency [12,13]. Despite this, our sample described a large number of subjects with GHD and provided information on the neonatal period, which is not always readily available. It is desirable that during all endocrinological evaluations of children with short stature, attention is paid to these signs that, although unusual, may occasionally be associated with idiopathic GHD.

## 5. Conclusions

In our cohort of pediatric patients with idiopathic GHD, the prevalences of neonatal signs and symptoms of GHD were low, except for neonatal hypoglycemia observed in 27.9% of the analyzed patients. Although these are not specific signs of idiopathic GHD, it is beneficial to investigate this information in the medical history during the clinical assessment of the child.

## Figures and Tables

**Table 1:** Prevalences of neonatal GHD signs in our cohort of children with idiopathic GHD.

	GHD Group (n = 190) n (%)	Control Group (n = 190) n (%)	*p*-Value
Hypoglycemia	53 (27.9%)	19 (10.0%)	<0.001
Hyperglycemia	17 (8.9%)	18 (9.5%)	1.000
Prolonged jaundice	37 (19.5%)	39 (20.5%)	0.898
Cholestasis	4 (2.1%)	4 (2.1%)	1.000
Cryptorchidism	6 (3.2%)	8 (4.2%)	0.785
Micropenis	8 (4.2%)	10 (5.3%)	0.809
Neonatal hypotonia	12 (6.3%)	11 (5.8%)	1.000
Failure to thrive	17 (8.9%)	17 (8.9%)	1.000

## Data Availability

The original contributions presented in the study are included in the article, further inquiries can be directed to the corresponding author.
